# Artificial Intelligence Based Approach for Classification of Human Activities Using MEMS Sensors Data

**DOI:** 10.3390/s23031275

**Published:** 2023-01-22

**Authors:** Yusuf Ahmed Khan, Syed Imaduddin, Yash Pratap Singh, Mohd Wajid, Mohammed Usman, Mohamed Abbas

**Affiliations:** 1 Department of Electronics Engineering, ZHCET, Aligarh Muslim University, Aligarh 202002, India; 2 Department of Electrical Engineering, King Khalid University, Abha 61411, Saudi Arabia; 3 Electrical Engineering Department, College of Engineering, King Khalid University, Abha 61421, Saudi Arabia; 4 Electronics and Communication Department, College of Engineering, Delta University for Science and Technology, Gamasa 35712, Egypt

**Keywords:** Bi-LSTM network, classification algorithm, deep learning, deep neural network, human activity recognition, machine learning, MEMS sensors, mobile & wearable devices, recurrent neural network

## Abstract

The integration of Micro Electronic Mechanical Systems (MEMS) sensor technology in smartphones has greatly improved the capability for Human Activity Recognition (HAR). By utilizing Machine Learning (ML) techniques and data from these sensors, various human motion activities can be classified. This study performed experiments and compiled a large dataset of nine daily activities, including Laying Down, Stationary, Walking, Brisk Walking, Running, Stairs-Up, Stairs-Down, Squatting, and Cycling. Several ML models, such as Decision Tree Classifier, Random Forest Classifier, K Neighbors Classifier, Multinomial Logistic Regression, Gaussian Naive Bayes, and Support Vector Machine, were trained on sensor data collected from accelerometer, gyroscope, and magnetometer embedded in smartphones and wearable devices. The highest test accuracy of 95% was achieved using the random forest algorithm. Additionally, a custom-built Bidirectional Long-Short-Term Memory (Bi-LSTM) model, a type of Recurrent Neural Network (RNN), was proposed and yielded an improved test accuracy of 98.1%. This approach differs from traditional algorithmic-based human activity detection used in current wearable technologies, resulting in improved accuracy.

## 1. Introduction

Recent advancements in Micro Electronic Mechanical Systems (MEMS) sensor technology and Artificial Intelligence (AI) have made human activity recognition (HAR) possible with high accuracy. A series of MEMS sensors and AI techniques are used to detect body motions to deduce critical information about it [1], such as the activity patterns of the user. The HAR applications vary from entertainment to the defense industry such as sports analytics, gaming, healthcare, smart homes, space exploration, personal fitness tracking, remote tracking, enhanced manufacturing, security, etc [1,2]. For example, for space exploration purposes a comprehensive/compact activity recognition system could be built on a space rover. The scientists would then be able to track its motion status, which is a vital piece of information. Another scenario could be, where a patient needs to be constantly monitored due to some diseases like diabetes, high blood pressure, high cholesterol, etc., therefore, tracking their motion activities like walking, jogging, running, cycling, etc. can provide feedback to them or their caregiver. The movement of a person can be tracked with the use of smart bands, mobile phones, and wearable devices. With such electronic devices in the market, users can access an extensive range of sensors for a wide spectrum of applications in both their professional and personal lives. Due to people’s increased awareness of their health, exercising and tracking their sleep have become fashionable trends for health enthusiasts [3]. By collecting behavioral data from these sensors, researchers are addressing needs in the medical & healthcare sectors and smart homes [4]. Various industries and technology giants also benefit from data collected in this way because it directs their research efforts to develop future products that can be released to the market.

There are a variety of sensors that can sense movements, namely, video cameras, wearable physiological sensors, motion sensors, RADAR [5], acoustic sensors [6], Echo (Amazon Echo, 2018), everyday objects (such as HAPIfork, 2018), food scales (SITU-The Smart Food Nutrition Scale); additionally, IR motion sensors, magnetic sensors, and other ambient sensors have also been employed extensively for motion activity recognition [7]. These devices are compact, cheap, and have fast processing/computing capabilities [8]. With wireless sensor networks [9], wearable devices (e.g., smart watches/fitness bands, body-worn sensors, MEMS Sensors, smartphones, etc.) can gather and transmit real-time data from different locations on the body (e.g., head, chest, upper arm, forearm, leg, etc.). HAR systems based on video cameras can be used for many different security applications, but they have many challenges involving with regards to privacy and space in smart environments. People apart from the target/ Other people, such as caregivers or family members may also be recorded by the device. The misuse of such videos contributes to security concerns and infringes upon their privacy, which is deemed unacceptable. Nonetheless, the sensors which are, wearable MEMS sensors, eliminate the security and privacy concerns related to the monitoring of activities [4]. MEMS sensors that are present in smartphones and wearable devices can be used to extract information by processing the data from these sensors. Accelerometers are also frequently employed for HAR along with gyroscopes, as they have shown improved recognition performance when used together [10]. As smartphones have developed, they have opened up previously unimaginable possibilities for monitoring and interacting with human subjects in real-life settings. Latest smartphones are programmable, equipped with numerous integrated MEMS sensors, large & high-resolution touch displays, faster & better CPUs, prolonged battery lives, greater storage memory, and wireless connectivity to external sensors/devices, as a result, they are widely used [11]. Moreover, there are wireless technologies that transmit data from the user’s body to a storage device located remotely. This allows new types of decision support systems to be developed, and data can be displayed on a device like a computer server. Despite the benefits of increased privacy and security, wearable sensors also present some challenges/obstacles, including intraclass variability, interclass similarity, class imbalance, and determining the actual start and end times of activities [4].

Data collected from sensors can be used to train a number of ML classifiers [12] which include Support Vector Machines (SVMs), Hidden Markov Models (HMMs), Dynamic Bayesian Models (DBMs), Random Forests (RFs), Decision Trees (DTs), etc. In order to utilize ML algorithms features need to be extracted from the collected data, even though the data size need not be enormous [13]. Traditional ML has been revolutionized by Deep Learning (DL) and has enhanced performance in many domains, some of which include image recognition, object detection, speech recognition, and natural language processing (NLP) [14]. With the help of ML & DL, HAR can be significantly improved in terms of performance and robustness, which enables it to be used for a variety of wearable sensor-based applications. In various applications, DL has been successful mainly because of two reasons. First, a DL algorithm is capable of automatically learning robust features from raw data sets for particular applications, while traditional ML methods engineer features using expert domain knowledge, the process is often very time-consuming and requires a great deal of expertise. Among DL models, recurrent neural networks (RNN), convolution neural networks (CNN), long short-term memory (LSTM), autoencoders, etc. are the most commonly used. Using deep neural networks (DNN), raw signals can be effectively analyzed with minimal domain knowledge. As a second benefit, DNN can be used to approximate practically any function, provided that they are dense enough and that there is sufficient observational data to do so [15,16,17]. As a result of their expressiveness, DL-based applications have grown substantially. The results of DL have been encouraging, but there remain several challenges and obstacles. These challenges include the need for large amounts of data, high computational requirements to run complex neural networks, and interpretability [18].

**Figure 1:**
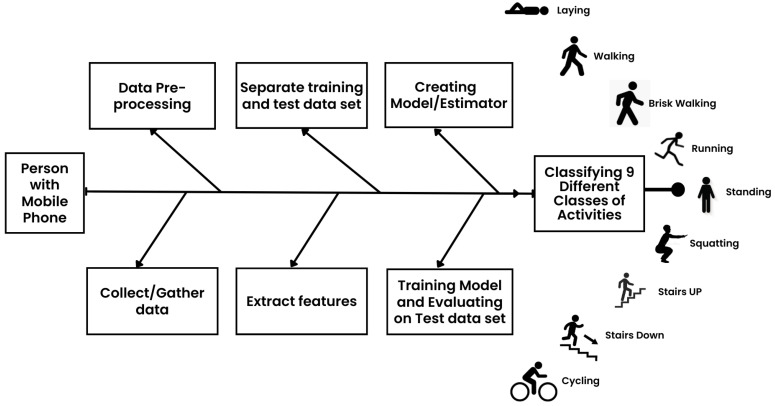
Steps for Human Activity Recognition.

In this work, after analyzing the various ML models, we put forward a Bi-LSTM DNN model for the classification of the said 9 motion activity classes as depicted in Figure 1, these activities are classified as activities of daily living (ADL). As part of our study, we aim to distinguish between these activities of daily living. Our focus is on HAR using embedded MEMS sensors. The Bi-LSTM DNN model uses the data recorded from MEMS sensors either separately or collectively to acquire information like acceleration, magnetic field, orientation, and angular velocity about all three axes (i.e., x, y, and z axes respectively). These MEMS sensors are not only cost-effective but they are also integrated into nearly every smartphone on the market today [19]. This study covers the following areas and has the following contributions:

(a) Rigorous experiments were conducted to prepare an extensive dataset of 9 different human motion activity classes which include (*a*) Laying Down, (*b*) Stationary, (*c*) Walking, (*d*) Brisk Walking, (*e*) Running (*f*) Stairs Up (*g*) Stairs Down (*h*) Squatting and (*i*) Cycling, the prepared dataset was then used for training and testing purposes for the ML and DL model(s). A detailed explanation is provided in Section 3.1.
(b) Dataset prepared through these experiments was then used to train various ML and DL model(s) as specified in Section 3.2.
(c) By combining an auto-labeling module with a DNN that uses Bi-LSTM structures, a supervised DL framework is designed, constructed, and proposed, which efficiently uses the extensively prepared dataset to achieve maximum HAR accuracy of 98.1%.
(d) The proposed DNN Bi-LSTM-based model was then tuned by varying several model parameters to conclude the best possible model (hyperparameter tuning). Various parameters like training & testing time, and size of the trained network were also observed for the different cases (parametric analysis), as elaborated in Section 3.3.
(e) Comparative analysis has been performed on the WISDM dataset, which is a publicly available dataset, Section 5 describes it in detail.

The manuscript proceeds as follows, Section 2 provides a comprehensive literature review on HAR, Section 3 describes the methodology used towards HAR, the results achieved are discussed in Section 4. A comparative analysis has been provided in Section 5. Finally the conclusion and future scope of the work is discussed in Section 6.

## 2. Literaure Survey

HAR is not something that is new to the researchers’ interest. It was in the 1990s when some [20] started exploring the field. But due to less conception of wearable devices at that time, good results were not seen. With the rapid development of wearable technology in the 21st century combined with the fast conception of wearable devices triggered the growth of HAR. This is mainly because of the proliferation of handheld devices with multiple built-in sensors. There are numerous ML and DL methods that can be used for classifying human activities, but utilizing them in a way to get more accurate results still needs to be worked upon. Modern devices are packed with a variety of sensors, like Accelerometer, Gyroscope, Magnetometer, but the accelerometer is still the most reliable. The work by Prasad et al. [12] using just an accelerometer and still getting good accuracy explains how powerful results can be achieved using just a simple sensor. The aim was to identify the six basic fundamental human activities, namely, walking, brisk walking, standing, sitting, and going upstairs or downstairs. They focused on utilising the accelerometer present in smartphones to detect the exercises by using a DL method naming Convolutional Neural Network (CNN). Their paper supports the implementation of a two-dimensional CNN model. It was found that the trained model was capable of classifying human activities with an accuracy of 89.67%. The approach to get much better accuracy is an open challenge in the work.

Some researchers extend the use of sensors to more than one sensor. Ronao et al. [21] used smartphones to collect the data for Human Activity Identification [22]. The data set was collected using the readings of accelerometer and gyroscope at a frequency of 50 Hz. The correct feature subset was collected using random forest variable importance measures. Six activities were classified, including walking, going upstairs, going downstairs, sitting, standing, and lying using a two-stage Hidden Markov Model (HMMs). They utilise the best from HMM- Gaussian Mixture Model (GMM) and used both of them separately. The use of GMM was to model the picked features and HMM to model the temporal reliance among actions. After analysing the results computed from two together-stage HMM, ANN, Decision Tree (DT), and Naive Bayes (NB), it was noted that the two together-stage HMM-GMM model performed best. Some researchers tried to fetch data using more complex ways, but the practical implementation of their ways is a problem to tackle, like, the work by Krishnan et al. [23] was to implement and collect the data by placing an accelerometer on the thighs of a subject, but when the data was tested, it lacked that accuracy and it did not perform well for activities like walking, sitting, lying down, etc. So, they conclude that multiple sensors are required to get the best out of the model. A higher degree of accuracy can be achieved by this, but in reality, it is really inconvenient to collect the data by placing many sensors on the body of the user.

As more researchers started working on HAR, different methods started to get utilised to maximise the accuracy and reduce the time to establish the classifier. Qi et al. [24] proposed to classify human action using a smartphone in a much fast way. They focused on providing an amazingly fast and powerful Deep Convolutional interconnected system form (FR-DCNN) for action recognition utilising a mobile phone. The experiment was performed on 12 complex data sets, which predicted that the FR-DCNN model is a high-quality design for fast calculation and extreme accuracy recognition. The MATLAB app on the smartphone was utilized for computing the activity readings. The time required by the FR-DCNN model to conclude the action was just 0.0029 seconds in a connection to the internet, accompanying 95.27% accuracy. Concurrently, only 88 seconds were required to base the DCNN classifier on the compressed dataset, resulting in a reduced accuracy deficit of 94.18%. It was completed later by instructing the consumers to record the 12 exercises by transferring the mobile phones established on the waist. HAR also started as a major breakthrough in medical applications. The work by Ali et al. [25] stated that one person collected the acceleration data using a mobile phone for a couple of days, to classify ADL into activities as stationary, light ambulatory, intense ambulatory, and abnormal classes. A J48 classifier is used to analyse the activities by feeding the collected data to a trained model. An accuracy of 70% was noted for each activity class and an accuracy of 80% was obtained by the model for stationary activities, and can easily differentiate between correlated activities like sitting on a chair and standing. Their work is remarkable and can have many amazing utilization in the medical field for monitoring purposes. Their work opens the door for more advance techniques to increase the accuracy of prediction.

There’s always a question as to which method or classifier to use in order to efficiently utilise the data collected by the user, so researchers did a comparative analysis of various models of DL. The research carried out by Hammerla et al. [26] tried to compare the different models of DL namely DNN, CNN, and RNN on some existing data sets of Opp, PAMAP2, and DG. They also compared two different variations of RNN that are deep forward LSTMs and bi-directional LSTMs. CNN got the highest accuracy on the PAMAP2 data set at 93.7%, while the LSTM and the b-LSTM classifiers got the maximum accuracy of 76% and 92.7% on the DG and OPP data sets, respectively. Their work claimed that one should rely more on RNNs when the activities are short-timed, but if the activities are long-term, then CNN is the best to work with. The question of which polling or sampling rate one should collect the data to utilise it effectively was carried out by a study of Maurer et al. [27] They used an accelerometer for the collection of data and observed how the accuracy is behaving when the sampling rate is varied from 10 Hz [28] to 100 Hz [29]. After checking the accuracy at different sampling rates, it was seen that no significant change occurred in accuracy as a function of sampling rate above 20 Hz. They stated that the more important thing to focus on is the placement of the accelerometer while collecting the data. He et al. [30] after numerous observations claimed that it’s best to place the accelerometer in the trousers pocket, alternatively many works suggest wearing it on the wrist [31], or belt [32], or in the bag carried by the user [27]. Their work concluded that the position of the accelerometer depends upon the type of readings one wants to calculate for what type of activity.

Suwannarat et al. [33] worked on reducing the dimensions of data collected by the accelerometer and determining its impact on the DNN-based HAR. They put forward an architecture by minimizing the parameters in accordance with the sample size that needs to be fed to the DNN. The parameters had been reduced to half of their baseline values, only the XY axes acceleration data is utilized, and the sample period had been reduced from 8s to 4s. The classifier worked fine and got comparable or better results than the baseline classifier. The UCI HAR, the Real World 2016, and the WISDM were the data sets that were used for carrying out the experiments by them. The results obtained by their research are really important, as they can help in the reduction of memory consumption, time reduction, and overall resource utilization on a better scale. The model presented can have many implementations, especially on low-powered devices like a smartwatch. The number of survey articles on HAR has also increased significantly in the past years [34,35,36,37,38]. The survey by Lima et al [39] provides a complete roadmap on how the HAR has been developed in the past years by providing a brief history of HAR and related works. In addition, the authors present results from the perspective of inertial sensors embedded in smartphones, which are important aspects of HAR solutions.

Recent studies in the field of HAR have explored the DL domain in a more detailed way. The work by Wang et al. [40] provides the usage of CNN and LSTM altogether to get much better results. Ramos et al. [41] used RNN, LSTM and GRU to get real-time detection of human activities. A one-dimensional Convolutional Neural Network with a bidirectional long short-term memory (1D-CNN-BiLSTM) model was presented by Luwe et al. [42] which results in a much better accuracy of 94.17% to all other recent works in HAR using DL. All the models presented by these papers are tested on some popular publicly available datasets which are sometimes not up to the mark for real-time HAR detection. The work by Liu et al. [43] provides an in-house collected dataset CSL-SHARE (Cognitive Systems Lab Sensor-based Human Activity REcordings) to classify 22 different activities with more accuracy. The use of decision tree classifiers to sense the changes in pressure using MEMS built accelerometer to collect and store data is provided by Pardeshi et al. [44]. Recent works by Patange et al. [45] and Shewale et al. [46] provided us with the importance of vibrations, temperature and other parameters in health monitoring systems. All these researches will lead us to develop more smart and accurate devices which will change human health monitoring systems forever.

Table 1 summarises the literature survey on HAR-related work performed by various researchers. These were the pieces of work that motivated the flow of this paper. All the research carried out in HAR always leaves a question: how to improve the model and recognize the activities in a more fast, reliable, and accurate way. In this paper, we present a comparative analysis between various ML and custom-built DL models and identifying the model which gives the highest accuracy.

## 3. Methodology

Existing wearable technology in the market does not specifically “classify” human motion activities and does not utilize ML techniques [47,48,49]. They only determine if the user is active or inactive by using some algorithm. In this research, we present a ML/DL-based approach for HAR to further improve classification accuracy in comparison to previous works by using the prepared dataset from sensors commonly found in smartphones. This is a baseline-level technology being proposed, which can be combined with several other existing technologies to be more application specific. For example, by combining the ML-based HAR system with other sensors like Sp02, BPM sensors, etc., the system can find a use case in the healthcare or fitness industry.

Throughout daily lives, humans perform a wide range of activities that can be classified automatically. However, this work identifies a few basic nine human activities, as given in Table 2. Each class of human motion activity has been assigned a unique numerical value from ’0’ to ’8’, these numerical values are used to classify the activities using ML and DL models.

### 3.1. Dataset

Data is like fuel to ML models; it is a key step before training an ML model. Publicly available datasets are widely used these days for training purposes, but they are generally too perfect or sometimes do not portray real-world conditions, hence as a result the models trained in such datasets aren’t able to generalize to new data and give out wrong results when deployed and tested in real-world conditions [50]. Therefore, to train a generalized model, as well as evaluate the model objectively, we have prepared our own data set by performing a large number of experiments for each human motion activity class, so as to achieve good training and testing accuracy with the proposed ML and DL models. Data of nine human motion activity classes has been collected using mobile phone sensors. The data set prepared consists of different readings such as magnetic field, angular velocity, orientation, and acceleration from the built-in mobile phone sensors i.e., magnetometer, gyroscope, and accelerometer, given in Table 3, respectively in all axes (i.e., x, y and z). These sensors’ signals were sampled at 100 Hz for the purpose of storing data and digitally processing for each class of human motion activity. A sampling frequency of 100 Hz is commonly used in HAR tasks as it strikes a balance between the need for high-resolution data and the practical limitations of data storage and computation. This sampling frequency is fast enough to capture the most important features of human motion, yet still manageable in terms of data size and processing time. A sampling frequency of 100 Hz means that the sensor data is collected 100 times per second, which allows for the capture of fast and subtle movements. The time duration is To avoid class imbalances, the time durations of each class have been taken the same, also keeping this in mind through data collection. After the collection of data, pre-processing of the data was done where the initial segment and final segment values of the data were removed, which contained erroneous data due to the unsteady state of the mobile phone at the start and end of the experiment. The outliers were observed by plotting a boxplot and removed.

**Figure 2:**
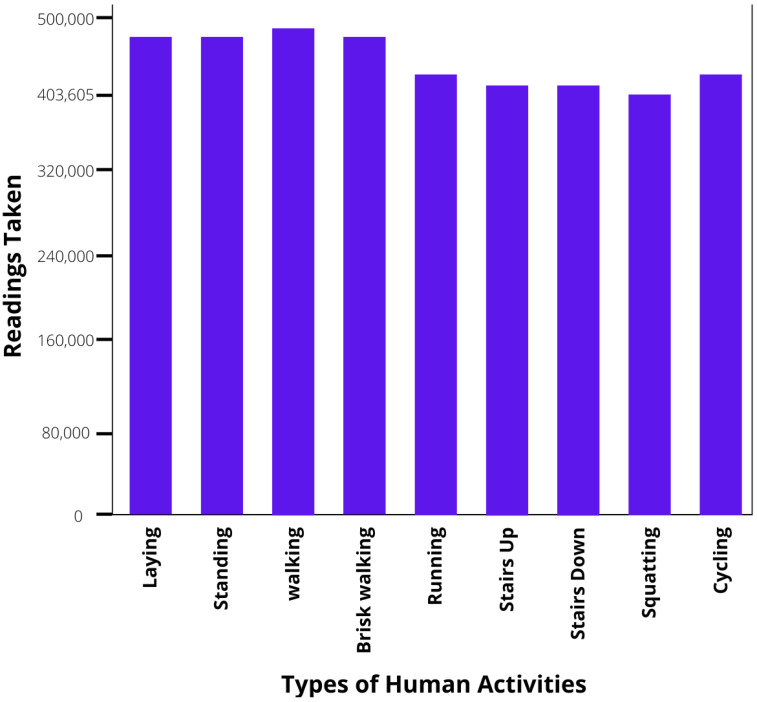
Data points per class in raw data.

The data in the raw format (data points per class) can be seen in Figure 2. The data points per class were made equal to avoid the class imbalance problem. After the pre-processing of data, the data was visually validated by plotting graphs of different parameters like magnetic field, angular velocity, orientation, and acceleration. Then the sensor readings were merged into one matrix file containing 12 columns (features for ML), which represent the magnetic field, angular velocity, orientation, and acceleration, in all three directions (X, Y, and Z). In the data matrix, there are 403,500 rows, of which 500 are considered as one experiment, so we have approximately 807 sets of experiments. Finally, the dataset was shuffled (to reduce variance and the problem of overfitting [51]) and divided into two segments: *(a)* 70% of the data set for the training and *(b)* 30% of data set for the testing, of ML and DL models. The training data set is used for training machine models, while the testing data set is used for evaluation purposes. The device specifications used for data collection and model training is given in Table 4.

A comparison of our prepared custom dataset with 12 existing publicly available datasets has been given in Table 5. This table contains detailed information about all these datasets, including the number of subjects, sampling rate, sample types, sensors, and classified activities.

### 3.2. Machine Learning for HAR

Preparation and pre-processing of the dataset were followed by the training of the various ML models *(a)* Decision Tree Classifier, *(b)* Random Forest Classifier, *(c)* K Neighbors Classifier, *(d)* Multinomial Logistic Regression, *(e)* Gaussian Naive Bayes, and *(f)* Support Vector Machine. These ML models have been briefly discussed as follows.

(a) Exactly as its name suggests, a *Decision Tree* represents a flowchart-like structure resembling a tree, where each internal node represents a test on an attribute, each branch represents a decision rule, and each leaf node (also known as a terminal node) exhibits the output. The parameters used for training the Decision Tree Classifier in our work are as follows, *min_samples_split*: this value indicates how many samples are required to split an internal node, *min_samples_leaf*: the minimum number of samples that must be at a leaf node. In each branch, the split point must leave at least min_samples_leaf training samples [63].
(b) *Random Forest Classifier* is a supervised ML algorithm that can be used to perform classification as well as regression problems, It aggregates several decision trees from various subsets of the dataset and improves predictive accuracy by taking the average. Its advantages include less train time than other algorithms and running efficiently on large datasets. The parameters used for training the Random Forest Classifier in our work are as follows, *n_estimators*: it specifies the number of trees in the forest, *criterion*: the quality of split is measured using this function, *Random State*: the randomness and bootstrapping is controlled with the help of this function [63].
(c) One of the simplest machine learning algorithms is the *K Nearest Neighbors (KNN) Classifier*, which uses proximity to classify or predict data points. A new case is placed into the category with the highest similarity to the available categories based on the similarity between the new case and the previously available cases. Since it does not learn from the training set immediately, it is also known as a lazy learner algorithm. Instead of learning from the dataset immediately, it stores it and later on performs a classification algorithm on it. The parameters used for training the KNN Classifier in our work are as follows, *algorithm*: the algorithm used to compute the nearest neighbours, possible values are ‘auto’, ‘ball_tree’, ‘kd_tree’, and ‘brute’, *n_neighbors*: specifies the number of neighbors to use by default for k-neighbors queries, *Weights*: function is used to make predictions, possible values are ‘uniform’, ‘distance’, and [callable] [63].
(d) *Multinomial Logistic Regression* is a modified version of logistic regression to incorporate multi-class problems as by default logistic regression performs binary classification (i.e., 0 or 1). The parameters used for training the KNN Classifier in our work are as follows, *Dual*: formulation with dual or primal components. The dual formulation is only implemented with the liblinear solver for l2 penalties. When the value of n_samples is greater than n_features, dual=False is preferred, *Tol*: stopping criteria tolerance, *C*: this value is the reverse of regularization strength and must be positive. Smaller values indicate stronger regularization, as in support vector machines, *fit_intercept*: it indicates whether the decision function should include a constant (a.k.a. bias or intercept) [63].
(e) Bayes’ theorem is applied with strong independence assumptions in *Gaussian Naive Bayes* probabilistic classification algorithm. Regarding classification, independence means that the presence of one feature value does not affect the presence of another. The parameters used for training the Gaussian Naive Bayes Classifier in our work are as follows, *var_smoothing*: for calculation stability, a portion of the largest variance of all features is added to variances [63].
(f) *Support Vector Machine (SVM)* plots each data item as a point in n-dimensional space (where n is the number of features), with each feature’s value being the coordinate value. Once the hyperplane differentiates the two classes very well, classification is conducted. After breaking down the multiclassification problem into multiple binary classification problems, the same principle is applied to the multiclass classification problem. In this technique, data points are mapped onto high-dimensional space and mutually linearly separated into two classes by breaking the multiclass problem into multiple binary classification problems. The parameters used for training the SVM Classifier in our work are as follows, *C*: this is the regularization parameter, must be positive, *Kernel*: an algorithm’s kernel type is specified here, *Degree*: Degree of the polynomial kernel function (‘poly’), *Gamma*: it is a kernel coefficient [63].

**Figure 3:**
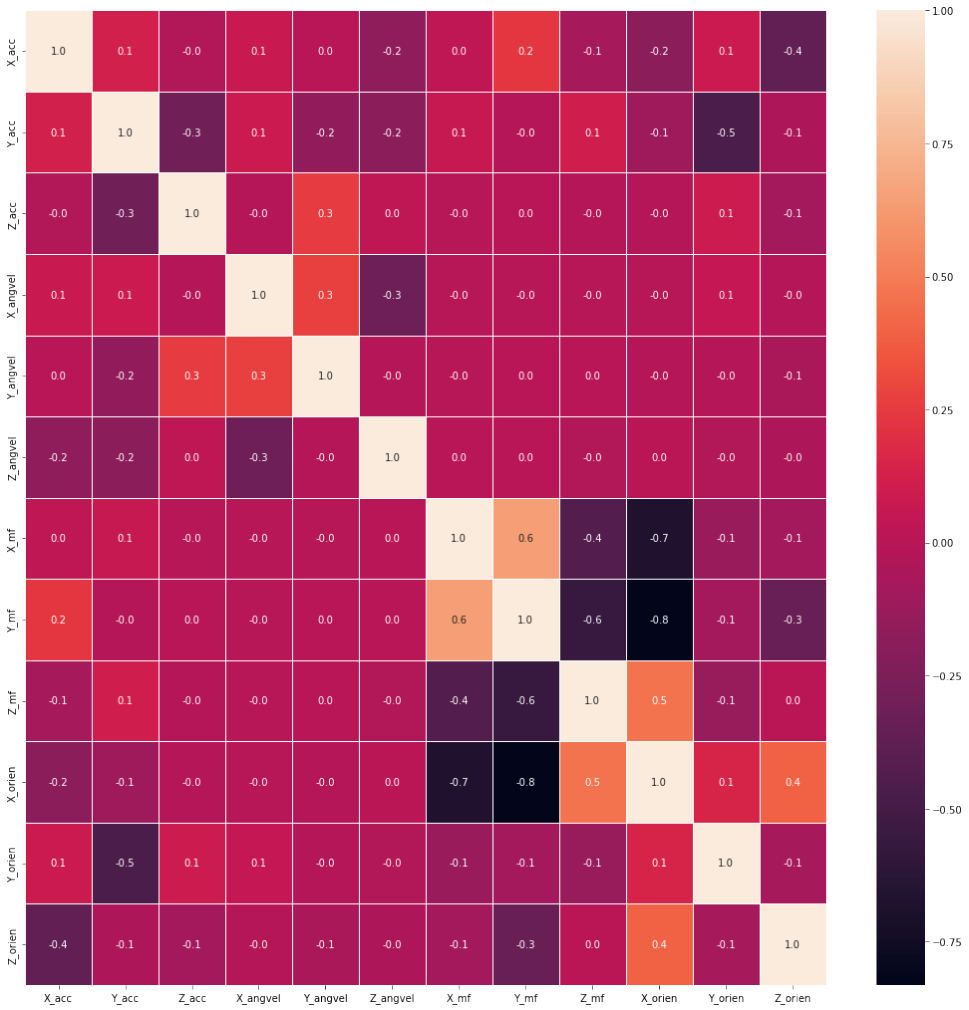
Correlation Matrix of the dataset obtained through experiments.

Values of all these parameters for the ML classifier are specified in Table 6, by the manual search method. In statistics, Pearson correlation coefficients measure linear associations between variables. The value ranges between −1 and 1, where −1 indicates a perfect negative correlation, 1 indicates a perfect positive correlation, and zero indicates no correlation between the two variables. Figure 3 shows the correlation matrix plotted for the dataset, it can be used to analyze the relation between our features used to train the ML models. The features used in our case are from Acceleration, Angular Velocity, Magnetic Field, and Orientation along all three axes (i.e., X, Y & Z), for instance, the abbreviation X_acc denotes the acceleration in the X direction, Z_orien denotes orientation along the Z axis and so on. Hence, as it can be observed from the correlation matrix (Table 7), our features are mostly distinct from each other, therefore all 12 of them have been utilized for training purposes.

**Figure 4:**
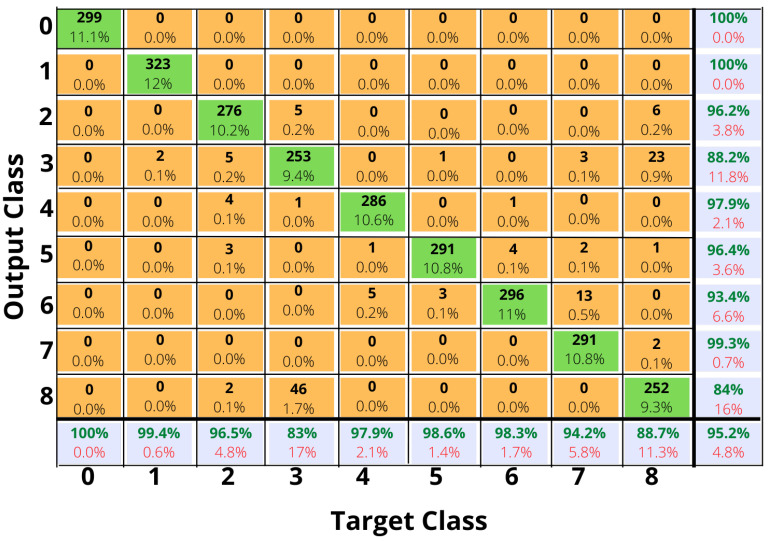
Confusion Matrix for HAR using Random Forest Classifier.

Using the six classifiers which have been discussed above, the ML models have been trained and tested for HAR accuracy, Table 8 shows the test accuracy of these models. The maximum accuracy which is achieved is 95% with the random forest classifier and multinomial logistic regression has the lowest accuracy at 67%. The confusion matrix for the maximum accuracy case using ML (random forest) is shown in Figure 4. Random forest classifier is predicting classes 0, 1, 5 and 6 with high accuracy (for classes numbering refer back to Table 2) and classes 3 and 8 are sometimes getting misclassified as the model is mispredicting these classes as the motion activity in these two classes is quite similar. As a further step towards improving the classification accuracy of human motion classes, DL for HAR is explored in the next Section. A model is developed to improve human motion classification accuracy.

### 3.3. Deep Learning for HAR

DL is a subset of ML, which utilizes the structure and functions of the human brain. In DL, an artificial neural network is used to compute complex calculations and classifications over large amounts of data. DL models are most commonly trained using the supervised learning technique. In supervised learning, a training data set is used to train the DL model to produce the desired outputs [64]. A classification-based supervised learning algorithm has been used in this work. Over time, the model learns from labeled inputs and adjusts its parameters based on the training data. In order to minimize the error, adjustment is made to the algorithm’s loss function until it reaches the desired level of accuracy. By adding more layers to the neural network, the accuracy value either increased or became saturated due to backpropagation [65]. In the backpropagation phase, the gradient and error calculations are determined. Once the gradients have been transmitted back to the hidden layers, the weights are adjusted. We continue determining the gradient and sending information back until we reach the input layer. As compared to traditional ML algorithms, like—shallow learning algorithms, it is a machine learning algorithm that reaches a performance plateau when we add more samples and training data to the network, deep learning algorithms like-DNNs, RNNs, LSTMs, etc, are much more scalable, and are able to solve more complex problems [66].

Deep Neural Networks (DNNs) are usually feed-forward networks, where data flows from input to output without going backward, and the connections between layers are constantly going forward and never touching the same node twice [67]. Since DNNs are forward-directed only they are stateless (have no memory), this stateless issue is addressed by RNNs. RNNs aren’t stateless, information flows back into the previous layers of the RNN network based on the connections between nodes that form a directed graph along a sequence. This enables information to persist across layers because each model depends on past events [68]. However, RNNs suffer from vanishing gradients/long-term dependency problems, where information disappears rapidly. This problem does not exist in Long Short-Term Memory (LSTM). LSTMs are a special breed of RNNs designed to learn dependencies over time, which helps them predict the future by recalling past patterns and memories [69]. Nowadays, LSTMs are widely used for Multilingual Language Processing, Machine Translation, Language Modeling, etc.

**Figure 5:**
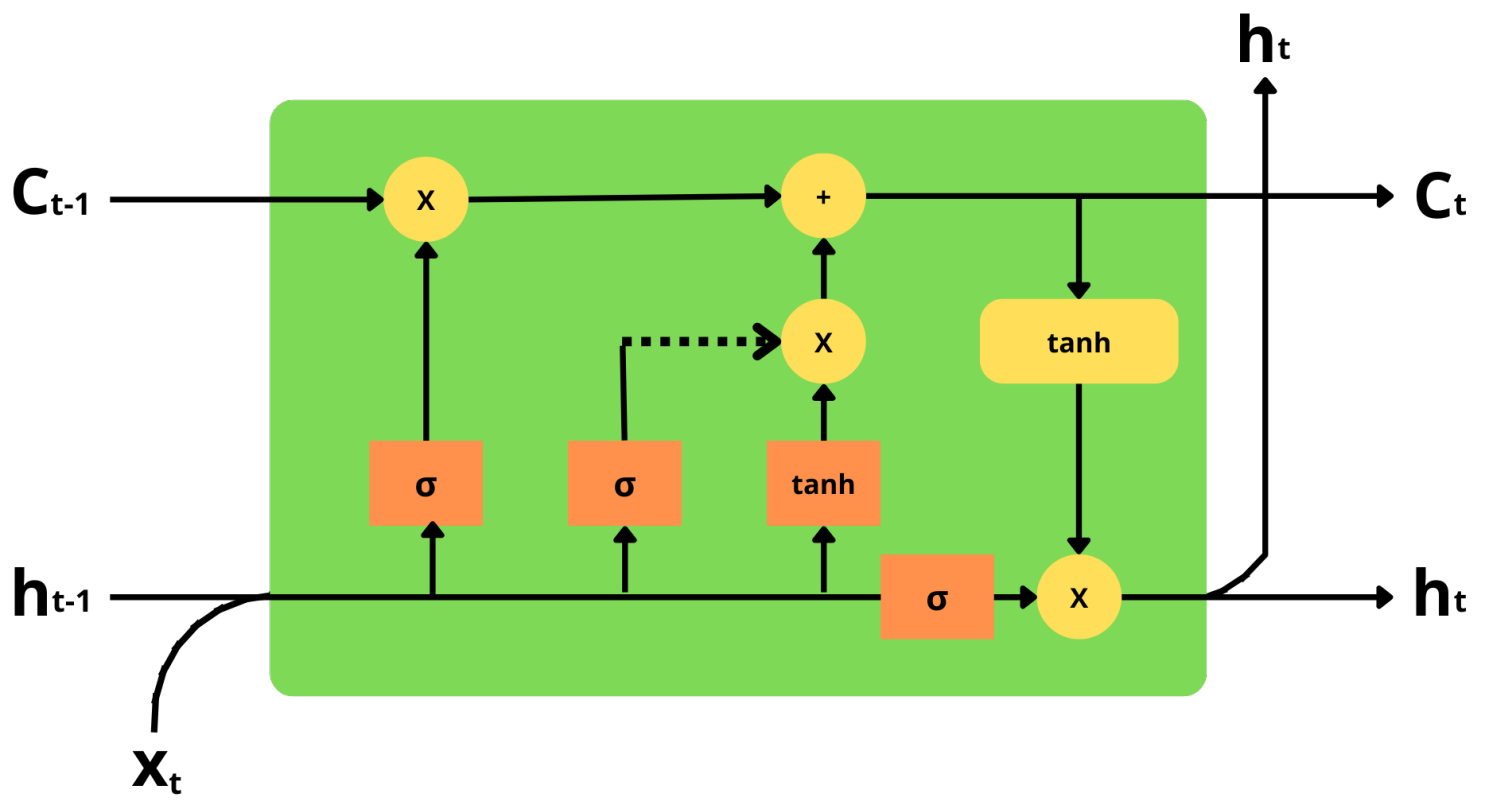
LSTM Architecture consists of 4 layers of neural network.

**Long Short Term Memory network, or LSTM,** is a special breed of RNN designed to learn dependencies over time, as shown in Figure 5. This network is extremely useful for a wide range of situations, and it is now widely used in different applications. LSTMs are specifically designed to overcome long-term dependency issues. In general, they have an innate ability to memorize information for long periods of time. There are a number of repeating modules in all recurrent neural networks. Standard RNNs consist of a single *tanh* layer as the repeating module. These chains are also common in LSTMs, but the repeating modules are different. Instead of a single layer, the LSTM consists of four layers of neural networks, each layer interacting in a specific way. LSTM weights can be dynamically modified without vanishing gradients or gradient expansion problems by modifying input, forgetting, and output thresholds [70]. In the field of technology, LSTM has a wide range of applications like speech recognition, picture recognition, robotics control, language translation, document abstraction, handwriting identification, and image analysis are only some of the applications for LSTM-based systems [71].

**Figure 6:**
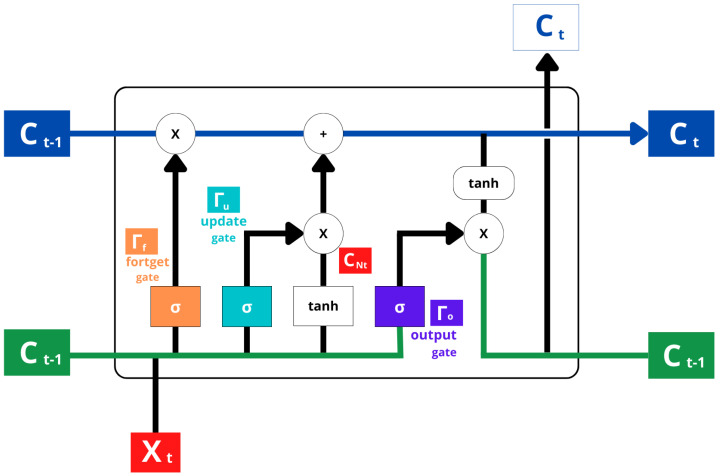
Bi-LSTM Architecture that takes input in both forward and backward directions.

**Bidirectional LSTM, or bi-LSTM network** is comprised of two LSTM networks. A forward-processing input is received from one and a backward-processing input is received from the other, as shown in Figure 6. The Bi-LSTM model extends the LSTM model based on forwarding calculation. The LSTM model can only predict subsequent units based on previous units, whereas the Bi-LSTM model can predict both from the front and the back. Traditionally, Bi-LSTM, RNN structures have been divided into two types: a forward RNN that is used for previous data, and a reverse RNN that is used for future data. Because of its structure, Bi-LSTM can always access previous and next information. It generally outperforms one-way LSTM in data with a heavy dependence on two-way information [72].

### 3.4. Architecture of the Proposed DL Model Using Bi-LSTM Neural Network for HAR

**Figure 7:**
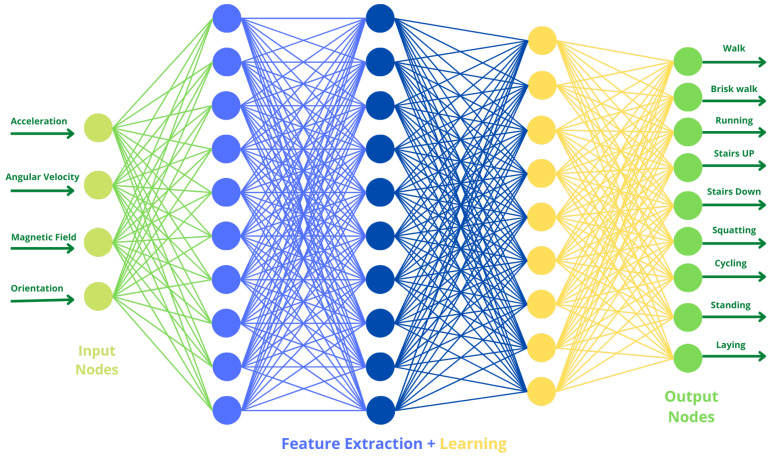
Deep Learning Model using Bi-LSTM for HAR.

We have made our own DL model using the Bi-LSTM network, the architecture of Bi-LSTM can be seen in Figure 6. As shown in Figure 7, the proposed Bi-LSTM model starts with the sequence input layer, with a value ‘12’ set as the input size for the sequence input layer (since we have total of a 12 features consisting of acceleration, angular velocity, magnetic field, and orientation in all three directions, i.e., x, y, z). Followed by the input sequence layer we have used the Bi-LSTM layer in which the number of hidden layers is set to ‘90’ (we get this number by analyzing the time taken, accuracy, and weights of our model on different values of the number of hidden layers from ‘10’ to ‘110’ at a gap of 10). Detailed analysis of the model on different numbers of hidden layers is given in Table 9. The model hyperparameter (number of hidden layers) has been varied from 10 hidden layers to 120 hidden layers and a maximum test accuracy of 98.1% has been observed for the case when 90 hidden layers have been chosen. Additionally, the number of training and testing elements, the training and testing time, the training and testing time per element, and the size of the trained network were observed. It can also be analyzed that by increasing the number of hidden layers, improvements in testing accuracy is observed until 90 hidden layers, after which the accuracy starts to decrease, and the size of the trained network also increases with the increase in the number of hidden layers as the network becomes more complex.

In addition to the number of hidden layers, the proposed Bi-LSTM model incorporates the following parameters [73]:

(a) Bi-LSTM can output two output modes, namely, *‘sequence’* & *‘last’*. Sequence outputs the entire sequence and last outputs the end of it. Since we only need the sequence’s final step, we selected ‘last’ [74].
(b) State activation function of Bi-LSTM model has two activation functions *‘tanh’* & the *‘softsign’* functions for updating the hidden layers. We used ‘tanh’ as the weights and bias are updated more frequently when using the ‘tanh’ function due to its high derivative [74].
(c) There are two types of gate activation functions available, namely, *‘sigmoid’* and *‘hard-sigmoid’*. We have selected ‘sigmoid’ function as ‘hard-sigmoid’ performs worse than ‘sigmoid’ [74,75].
(d) Input weight initializers, initialize input weights, based on the following options, *‘glorot’*—create weights such that every layer’s activation variance is the same, *‘he’*—used in order to achieve a variance of approximately one, *‘orthogonal’*—used to prevent gradients from exploding and disappearing, *‘narrow-normal’*—starting with an average of ‘0’ and a standard deviation of ‘0.01’ input weights randomly selected from a normal distribution, *‘zeros’*—weights are initialized to zeros, *‘ones’*—weights are initialized to ones. We have selected ‘glorot’ as our input weights initialization function to maintain a smooth distribution for both forward and backward propagation [74].
(e) Recurrent weights initializer serves as an initialization function for the recurrent weights. There are the same options as in the input weights initializer that we discussed earlier. We have selected ‘orthogonal’ as our recurrent weights initialization function because the gradient descent can achieve zero training error in a linear convergence rate for orthogonal initialization [74].
(f) Input weights learn rate factor is multiplied by the global rate of learning in order to determine the input weights’ learning rate. To make the learning rate factor equal to the global rate of learning, we set it to ‘1’ [74].
(g) Recurrent weights learn rate factor is the learning rate factor of the recurrent weights and multiplying it by the global rate of learning gives us the recurrent weights of the layer. For the recurrent weights, we set the learning rate factor to ‘1’ to make it equal to the global rate of learning [74].
(h) Input weights layer-2 factor is used to reduce the possibility of overfitting, layer-2, it is a data link layer, regularization keeps weights and biases small. For the value 1, the input weights of data link layer factor matches the current global data link layer regularization factor [74].
(i) Bias learn rate factor is a non-negative scalar or 1-by-8 numerical vector that specifies the learning rate for biases. A learning rate factor of ‘1’ is applied to biases to make them equal to the global rate of learning [74].
(j) Bias layer-2 factor is a non-negative scalar is specified as the regularization factor for the biases based on the data link layer regularization. By multiplying this factor to the global factor data link layer regularization determines the data link layer regularization for biases in the layer. It’s set to zero because it doesn’t need to be equal to global data link layer regularization factor [74].
(k) In Bias initializer, one of the following functions is used to initialize the bias, *‘unit-forget-gate’*—creates the forget gate bias with ‘1’, the other biases with ‘0’, *‘narrow-normal’*—starting with an average of ‘0’ and a standard deviation of ‘0.01’ input weights randomly selected from a normal distribution, *‘ones’*—weights are initialized to ones. We used ‘unit-forget-gate’ to decide what information should be paid attention to and which should be ignored [74].

All the above defined parameters of Bi-LSTM layer is summarized in Table 10.

**Figure 8:**
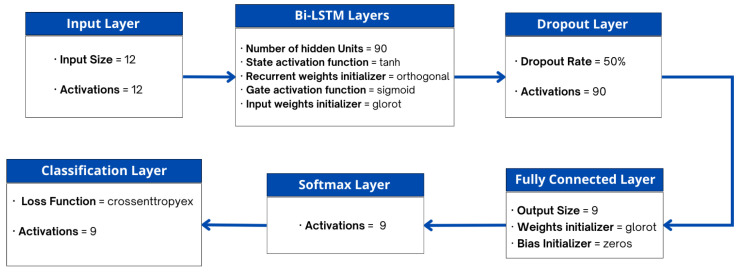
Architecture of the proposed Bi-LSTM Model.

To prevent neural networks from overfitting, we have a dropout layer after the Bi-LSTM layer, in each iteration, it randomly drops neurons from the neural network. The dropout layer in the proposed model has a probability of *0.5*, because the common value is a probability of *0.5* for retaining the output of each node in a hidden layer [76]. Followed by the dropout layer we have a fully connected layer, as a fully connected neural network is used to classify data after feature extraction [77]. A softmax layer is added after fully connected layer in our model, it is widely used for multi-class classification problems requiring classifications on more than two labels. Lastly, we have classification layer, which has a loss function as *crossentropyex*, which is used to compute the cross-entropy loss during classification and weighted classification tasks. The architecture of the proposed Bi-LSTM model is summarized in Figure 8.

## 4. Performance Evaluation and Results

In the training data set, information from the accelerometer, gyroscope, and magnetometer was used to build a DL model and train the parameters in Bi-LSTM. Our understanding that neural networks are efficient in solving optimization problems makes it possible to answer the question of how errors are evaluated for sets of weights by training them. If we are unable to predict the right output, a loss occurs based on how much the model deviates from the actual result. It is widely accepted that mean square error and cross-entropy are the two most widely used loss functions when neural networks are trained. In order to improve classification models, cross-entropy loss functions (CELFs) are generally used [78]. We also used CELF to adjust the weights of our models during training on the last classification layer. CELF can be calculated as follows

(1) $$Loss=-\sum_{i=1}^{OutputSize}y_{i}\cdot log\hat{y_{i}}$$

In Equation (1), $y_{i}$ represents the ith actual value, $\hat{yi}$ represents the neural network’s prediction for ith value, and OutputSize represents the number of classes [79]. Mean square errors (MSEs) are often used in regression analysis. But they cannot be used to assess classification problems and can be calculated by squaring the predicted values and the true values [19].

For the purpose of assessing the performance of our model, the metrics, Accuracy, Precision, Recall, F1 Score, confusion matrix, and loss/accuracy metrics are used [36,80,81,82]. The definitions of these matrices are 

*Accuracy*: An accuracy measure is calculated by the ratio of the number of predictions made to the number of classifications that are correctly predicted.

(2) $$Accuracy=\frac{Correct\;predictions}{Total\;predictions}$$

*Precision*: A sample’s precision is a measure of how many accurately identified positive samples are in relation to the total number of positive samples. which is defined as

(3) $$Precision=\frac{Accurate\;positive\;samples}{Total\;positive\;samples}$$

*Recall*: The recall of a model is a measure of how well a model finds all relevant cases within a data set. Mathematically, recall equals to the proportion of true positive samples to the summation of false negative samples and true positive samples.

(4) $$Recall=\frac{True\;positive}{True\;positive\;+\;False\;negative}$$

F1Score: The harmonic mean of precision and recall, also referred to as the balanced F score, is a combination of the accuracy and recall indicators’ findings.

(5) $$F_{1}Score=\frac{2\times Precision\times Recall}{Precision\;+\;Recall}$$

The value of these metrics for the maximum accuracy 98.1%, is given in Table 11.

**Figure 9:**
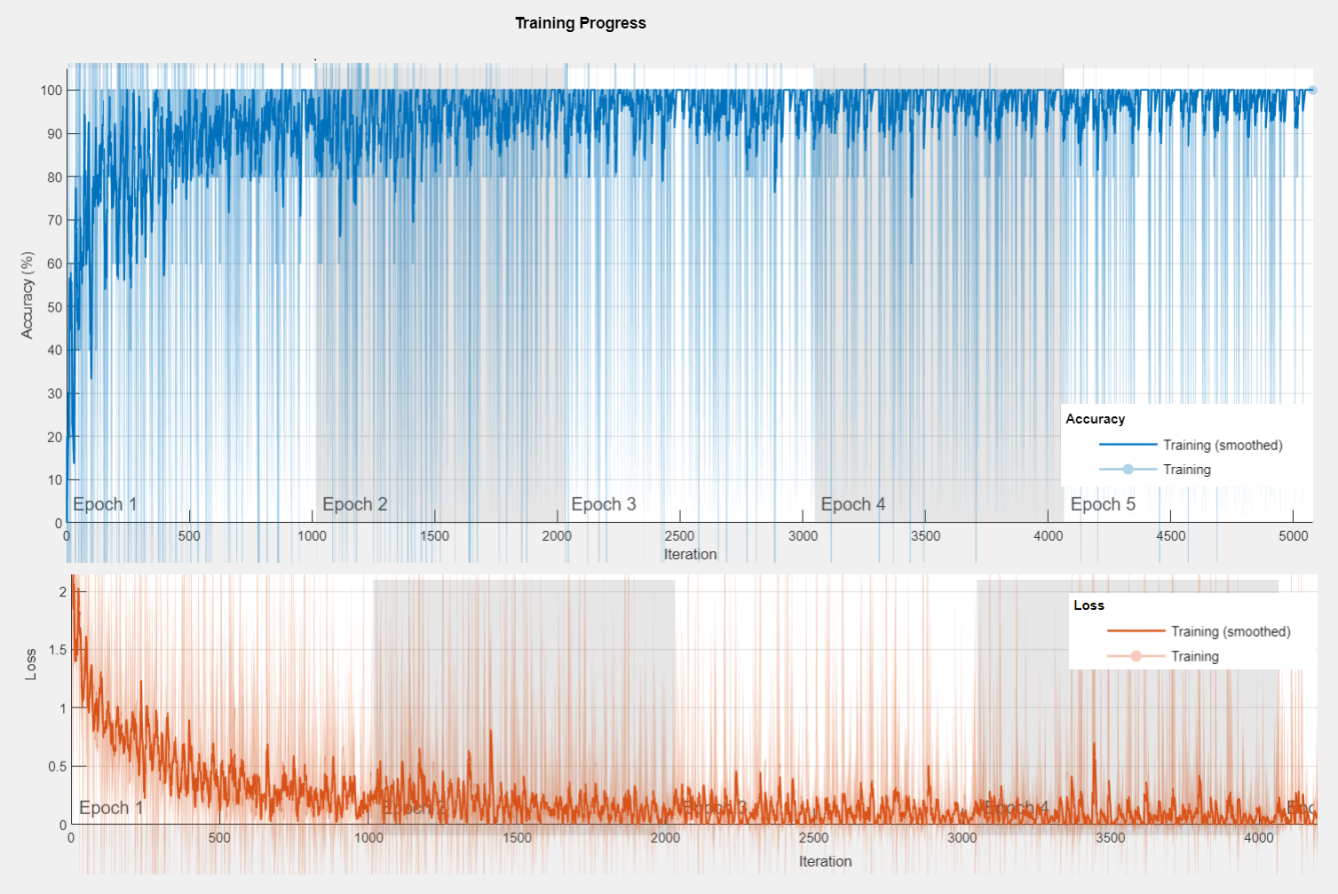
Accuracy and loss curve of maximum accuracy with the proposed DL model.

*Accuracy and loss map* [83]: As the neural network model is trained, the response to fluctuations in accuracy and loss is measured, as shown in Figure 9. A loss and accuracy value will be generated for each epoch. The accuracy and loss diagrams can be used to visually represent the network model’s training. The trend can be used to detect time abnormalities (like underfitting and overfitting) and perform real-time changes to see if the model was trained effectively and appropriately.

**Figure 10:**
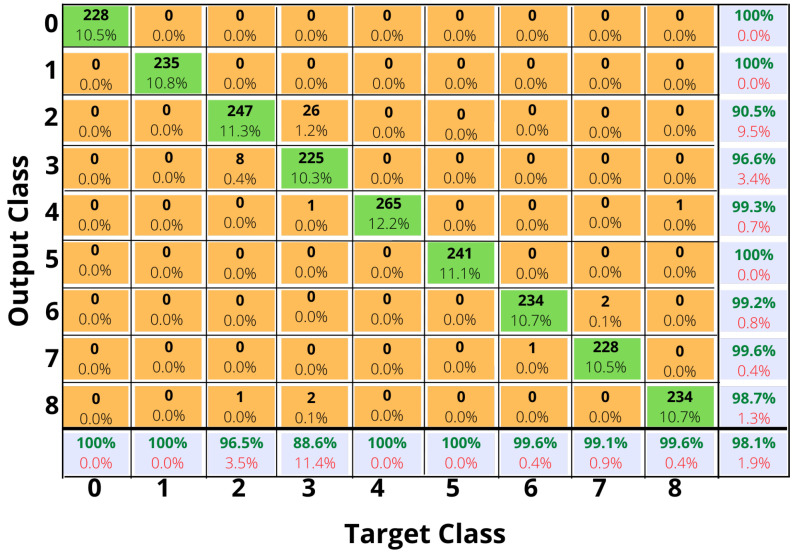
Confusion matrix for the proposed Bi-LSTM model for the maximum accuracy case, as seen in Table 9.

*Confusion matrix*: When our classification model makes predictions, the confusion matrix shows how it gets confused [82,84]. It summarizes the performance of classifiers by using a confusion matrix, as shown in Figure 10. In data sets with more than two classes or unequal numbers of observations in each class, classification accuracy alone could be misleading. In order to determine what types of errors our classification model makes, we need to calculate a confusion matrix.

A variety of ML models are used to evaluate HAR for the classes described in Table 2, including Multinomial Logistic Regression (MLR), Gaussian Naive Bayes (GNB), Decision Tree Classifier (DTC), Random Forest Classifier (RFC), K-Nearest Neighbour (KNN), and Support Vector Machine (SVM). As a result of our experiments, RFC achieved the best accuracy of 95%, compared to other ML models. The accuracy results of all ML models used are summarized in Table 8 and the confusion matrix for RFC is shown in Figure 4, illustrates the correct and incorrect predictions, as well as the accuracy for each human motion activity class, by the RFC. To achieve better results, DL is explored and a Bi-LSTM based model is proposed.

We conducted experiments with our proposed Bi-LSTM DL model, by varying the number of hidden layers from 10 to 120. As shown in Table 9, best overall accuracy of 98.1% is achieved for 90 hidden layers. The confusion matrix for the proposed Bi-LSTM model is shown in Figure 10, the values from ’0’ to ’8’ represent the human motion activities described in Table 2. In the confusion matrix, the accuracy and errors for different human motion activity classes are presented, it can be observed that most of the data points related to brisk walking are categorized as walking and vice versa. It can thus be concluded that it is difficult to differentiate between walking and brisk walking using sensor data. Therefore, walking and brisk walking have less accuracy than other human motion activities. Accuracy and loss curve for the maximum accuracy case (98.1%) is shown in Figure 9.

Overall, using different holdout percentages can provide a more comprehensive understanding of a model’s performance and its ability to generalize to unseen data. This information can be used to improve the model’s performance and to make more informed decisions about its deployment in practical applications. Holdout is where a portion of the data is set aside as a test set, while the remaining data is used for training. The holdout percentage refers to the proportion of the data that is set aside as the test set. A common approach is to use a holdout percentage of 20–30% for the test set, and the remaining data for training.

It is apparent from the Table 12 that different percentages of holdout result in different accuracy results; the highest accuracy is 98.1% if we split training and testing 70–30. It is crucial to do this analysis in order to properly evaluate the model, as the optimum split must be determined. If the testing data is kept small, we may not be able to evaluate the model properly. Similarly, if training data are kept less, the model will not train appropriately and will provide incorrect results.

## 5. Comparative Analysis

For evaluating our proposed Bi-LSTM, a comparative analysis has been done on a pre-processed data set released by the Wireless Sensor Data Mining (WISDM) Lab [61]. For the purpose of evaluating real-world human activity, this dataset was collected using the actitracker system. A total of 36 subjects were equipped with accelerometer sensors to collect data. The data set contains readings of 6 different human activities: walking, jogging, upstairs, downstairs, sitting, standing. We have provided a complete parametric analysis of WISDM dataset in the Table 13.

Our model performed quite well on WISDM dataset, after varying various hyperparameters our model was able to achieve an accuracy of 96.3%. A comparative analysis of our model with earlier studies on WISDM dataset was performed to determine its adaptability. The accuracy of our model against some of the latest works in the HAR domain is listed in Table 14.

## 6. Conclusions

In this study, multiple Machine Learning (ML) and a Deep Learning (DL) model(s) were utilized to classify nine different human motion activities, and a comparative study of the proposed model on the WISDM dataset with previous works on HAR is also presented. After experimenting with several ML models, including Random Forest Classifier (RFC), Decision Tree Classifier (DTC), K-Nearest Neighbors (KNN), Multinomial Logistic Regression (MLR), Gaussian Naive Bayes (GNB), and Support Vector Machine (SVM), the highest accuracy of 95% was achieved using the RFC. Furthermore, a DL model using Bidirectional Long-Short-Term Memory (Bi-LSTM) was proposed for HAR, which performed better than the other ML models. The proposed DL model employs a supervised deep learning framework based on Bi-LSTM and a Bi-LSTM-based neural network was constructed to handle sequential motion data, with a classification mechanism that is improved to identify fine-grained motion patterns based on features extracted from the dataset. Through hyperparameter fine-tuning, the proposed model achieved an accuracy of 98.1%. The experiment used mobile phone sensors to collect data and implementing a Bi-LSTM model for HAR resulted in significant improvement in classification. Therefore, the proposed Bi-LSTM model is found to be practical and useful based on evaluation results. Additionally, we compared the time taken, accuracy, and weights of the proposed Bi-LSTM model for different numbers of hidden layers.

In the future, we plan to investigate other machine and deep learning techniques for accurately identifying human activities from sensory, image, and video data. Further evaluations in different scenarios will be conducted to improve the algorithm’s reliability and efficiency. Additionally, the proposed Bi-LSTM model, with a test accuracy of 98.1%, can be implemented on various micro-controllers, micro-processors, FPGA boards, and other devices for prototyping and to validate these results via hardware as part of the development of Edge AI. After successful implementation, the cost of the product (a comprehensive HAR system) can be reduced by creating a custom chip for commercialization. This HAR system has potential applications in areas such as healthcare and surveillance. By adopting cloud-based techniques, smartphones, appliances, vehicles, computers, and other devices can be made more efficient, faster, and safer.

## Figures and Tables

**Table 1:** Summary of the literature review of the past work related to HAR.

Author, Year	Dataset	Purpose	Classification Techniques	Accuracy	Comments
Prasad et al, 2021 [12]	Self collected	Classification of 6 different classes of activities	Two dimensional CNN model	89.67%	Only accelerometer is used to collect data, accuracy can also be improved by other DL models.
Ronao et al, 2014 [21]	Self collected	Classification of 6 different classes of activities	HMM-GMM classifier	91.76%	The HMM-GMM model performed better than ANN, DT and NB
Krishnan et al, 2009 [23]	Self collected	Recognition of short duration hand movement	AdaBoost, HMM, k-NN	86%	Collecting data using large amount of sensors can increase accuracy but it is not feasible.
Qi et al, 2019 [24]	Self collected	Classification of 12 different classes of activities	FR-DCNN classifier	Normal dataset–95.27% Compressed dataset–94.18%	The proposed model performed really well with a fast speed and good accuracy.
Ali et al, 2020 [25]	Self collected	Classifying activities in stationary, light ambulatory, intense ambulatory and abnormal classes	J48 Classifier	stationary activities–80% other activities–70%	Their work can have implementation in medical field for monitoring purposes but a higher magnitude of accuracy is required.
Hammerla et al, 2019 [26]	Opp, PAMAP2 DG	Classifying 11 Activities of daily living	CNN, LSTM and b-LSTM	CNN–93.7% LSTM–76% b-LSTM–92.7%	This works claimed that CNN should be preffered for long-term activities and RNN for short-term
Maurer et al, 2006 [27]	Self collected	Comparing the impact of sampling rate and location of data collecting device on the accuracy	Decision tree	Highest accuracy–92.8%	No significant change in accuracy was noted above 20Hz sampling rate
He et al, 2008 [30]	Self collected	Classification of 4 different classes of activities	SVM model	92.25%	The position of accelerometer depends on the type of activity one wants to recognise.
Suwannarat et al, 2021 [33]	UCI HAR, the Real World 2016 and the WISDM	To create a light weight classification model	DNN based classifier	Comparitive or better accuracy than baseline classifier	The model presented can have many application specially in smartwatches.

**Table 2:** Assignment of numeric values to each human motion activity class.

HAR Class	Numeric Value	HAR Class	Numeric Value
Laying	0	Squatting	5
Stationary	1	Stairs-up	6
Walking	2	Stairs-down	7
Brisk-walking	3	Cycling	8
Running	4		

**Table 3:** Sensors and respective parameters read.

Sensors	Parameters Read
Accelerometer	Acceleration, Orientation
Gyroscope	Angular Velocity, Orientation
Magnetometer	Magnetic Field

**Table 4:** Device Specifications.

Purpose	Device	Specifications
Data collection	Smartphone	128 GB 6 GB RAM, Exynos 9825 (7 nm), Octa-core (2 × 2.73 GHz Exynos M4 & 2 × 2.40 GHz Cortex-A75 & 4 × 1.95 GHz Cortex-A55)
Model training	Laptop	11th Gen Intel(R) Core(TM) i5-1135G7 @ 2.40 GHz 2.42 GHz, 16.0 GB RAM

**Table 5:** Public human activity datasets for evaluation.

Dataset	Subject	Sample Rate (Hz)	Activity	Sample	Sensor	Reference
OPPORTUNITY	4	32	16	191,564	A, G, M	[52]
PAMAP2	9	100	18	64,173	A, G, M	[53]
DSA	8	25	19	75,998	A, G, M	[54]
MHEALTH	10	50	12	40,522	A, G, M	[55]
HHAR	9	100–200	6	366,038	A, G	[56]
Skoda	1	96	10	22,000	A	[57]
Daphnet Gait	10	64	2	49,942	A	[58]
UCI Smartphone	30	50	6	10,299	A, G	[22]
USC-HAD	14	100	12	41,998	A, G	[59]
SHO	10	50	7	20,998	A, G, M	[60]
WISDM v1.1	29	20	6	91,515	A	[61]
WISDM v2.0	36	20	6	248,653	A	[62]
**Our Custom Dataset**	**3**	**100**	**9**	**3,631,500**	**A, G, M**	-

A: Accelerometer; G: Gyroscope; M: Magnetometer.

**Table 6:** Machine Learning Parameters Summary.

Classifier Name	Parameter	Parameter Value
Random Forest	1. n_estimators 2. criterion 3. random state	1. 100 2. gini 3. 43
Decision Tree	1. min_samples_split 2. min_samples_leaf	1. 2 2. 1
Support Vector Machine	1. C 2. Kernel 3. Degree 4. gamma	1. 1 2. rbf 3. 3 4. scale
Gaussian Naïve Bayes	1. var_smoothing	1. $10^{-9}$
K Nearest Neighbours	1. algorithm 2. n_neighbors 3. weights	1. auto 2. 10 3. uniform
Multinomial Logistic Regression	1. dual 2. tol 3. C 4. fit_intercept	1. false 2. $10^{-4}$ 3. 1 4. true

**Table 7:** Correlation Matrix of the dataset obtained through experiments.

	X_acc	Y_acc	Z_acc	X_angvel	Y_angvel	Z_angvel	X_mf	Y_mf	Z_mf	X_orien	Y_orien	Z_orien
X_acc	1	0.1	0.0	0.1	0.0	−0.2	0.0	0.2	−0.1	−0.2	0.1	0.4
Y_acc	0.1	1	−0.3	0.1	−0.2	−0.2	0.1	0.0	0.1	−0.1	−0.5	−0.1
Z_acc	0.0	−0.3	1	0.0	0.3	0.0	0.0	0.0	0.0	0.0	0.1	−0.1
X_angvel	0.1	0.1	0.0	1	0.3	−0.3	0.0	0.0	0.0	0.0	0.1	0.0
Y_angvel	0.0	−0.2	0.3	0.3	1	0.0	0.0	0.0	0.0	0.0	0.0	−0.1
Z_angvel	−0.2	−0.2	0.0	−0.3	0.0	1	0.0	0.0	0.0	0.0	0.0	0.0
X_mf	0.0	0.1	0.0	0.0	0.0	0.0	1	0.6	−0.4	−0.7	−0.1	−0.1
Y_mf	0.2	0.0	0.0	0.0	0.0	0.0	0.6	1	−0.6	−0.8	−0.1	−0.3
Z_mf	−0.1	0.1	0.0	0.0	0.0	0.0	−0.4	−0.6	1	0.5	−0.1	0.0
X_orien	−0.2	−0.1	0.0	0.0	0.0	0.0	−0.7	−0.8	0.5	1	0.1	0.4
Y_orien	0.1	−0.5	0.1	0.1	0.0	0.0	−0.1	−0.1	−0.1	0.1	1	−0.1
Z_orien	−0.4	−0.1	−0.1	0.0	−0.1	0.0	−0.1	−0.3	0.0	0.4	−0.1	1

**Table 8:** Test accuracies of various ML models.

Model	Test Accuracy %
Multinomial Logistic Regression	67
Gaussian Naive Bayes	89
Decision Tree Classifier	93
**Random Forest Classifier**	**95**
K Neighbors Classifier	91
Support Vector Machine	93

**Table 9:** Detailed Parametric analysis of the proposed Bi-LSTM DL model.

No. of Hidden Layers	No. of Training elements	No. of Testing Elements	Total Training Time (sec)	Total Testing Time (sec)	Training Time per Element	Testing Time per Element	Testing Accuracy	Size of Trained Network (KB)
10	5084	2179	1274.9	26.26	250.77	12.05	90.5	1,143,306
20	5084	2179	366.73	24.68	72.13	11.33	95.1	1,143,364
30	5084	2179	743.61	8.38	146.26	3.85	95.7	1,143,387
40	5084	2179	432.96	24.9	85.16	11.43	96.9	1,143,465
50	5084	2179	412.32	8.61	81.1	3.95	97.06	1,143,522
60	5084	2179	408.23	8.45	80.3	3.88	97.29	1,143,557
70	5084	2179	371.23	8.42	73.02	3.86	95.96	1,143,647
80	5084	2179	336.83	9.18	66.25	4.21	96.65	1,143,758
**90**	**5084**	**2179**	**969.36**	**20.24**	**190.67**	**9.29**	**98.1**	**1,143,849**
100	5084	2179	376.1	8.27	73.98	3.8	97.15	1,144,000
110	5084	2179	342.52	8.07	67.37	3.7	97.4	1,144,137
120	5084	2179	417.89	9.65	82.2	4.43	97.43	1,144,315

**Table 10:** Bi-LSTM layer parameters for proposed DL model.

Parameters	Value/Function
Output mode	last
State activation function	tanh
Gate activation function	sigmoid
Input weights initializer	glorot
Recurrent weights initializer	orthogonal
Input weights learn rate factor	1
Recurrent weights learn rate factor	1
Input weights layer-2 factor	1
Bias learn rate factor	1
Bias layer-2	1
Bias initializer	unit-forget-gate

**Table 11:** Testing parameters of the proposed Bi-LSTM model.

Class	Precision	Recall	F1 Score
0	1	1	1
1	1	1	1
2	0.90	0.96	0.93
3	0.96	0.88	0.92
3	0.99	1	0.99
5	1	1	1
6	0.99	0.99	0.99
7	0.99	0.99	0.99
8	0.78	0.99	0.99

**Table 12:** Analysis for Different Train-Test (Holdout) percentages.

Train-Test (%)	Observed Accuracy (%)
60–40	96.5
65–35	97.2
**70–30**	**98.1**
75–25	97.9
80–20	95.5
85–15	97.8

**Table 13:** WISDM Dataset Raw-Data Statistics.

Parameters	Value
Number of examples	1,098,207
Number of classes	6
Missing attribute values	NONE
Walking	424,400 (38.6%)
Jogging	342,177 (31.2%)
Upstairs	122,869 (11.2%)
Downstairs	100,427 (9.1%)
Sitting	59,939 (5.5%)
Standing	48,395 (4.4%)

**Table 14:** Comparative analysis of proposed model with earlier works on WISDM dataset.

Reference	Algorithm	Accuracy (%)
Reuda et al. [85]	attrCNN-IMU	92.0
Ravi et al. [86]	Deep Learning Models	92.7
Zhang et al.[87]	HMVAN	93.1
Athota et al. [88]	CMFA	94.98
Athota et al. [88]	CGFA	84.35
Ullah et al. [89]	Stacked LSTM	93.13
Ordóñez, F. J. et al. [90]	LSTM	95.75
**Proposed Model**	**Bi-LSTM**	**96.30**

## Data Availability

Not applicable.
